# Dr. R. M. Swearingen

**Published:** 1898-10

**Authors:** 


### Society Notes.

For the Texas Medical Journal.

### Dr. R. M. Swearingen.

RESOLUTIONS OF RESPECT.—LAMAR COUNTY MEDICAL SOCIETY.

Died in Austin, Texas, August 7, 1898, our esteemed contemporary, State Health Officer Dr. R. M. Swearingen. While not a member of this Association, he was recognized throughout Texas as a man of marked medical achievements and of State renown, and we feel that we have lost a dear friend and adviser. Therefore be it resolved,—by the Lamar County Medical Association, assembled,

1st. That the medical profession of our State has lost one of its brightest minds and noblest hearts.

2nd. That the State, by his death, has lost a most vigilant and careful protector of its portals, he having zealously guarded its many entrances against epidemic and contagious diseases, and thereby on many occasions, prevented the wide spread of diseases, caused by the appearances of such contagions.

3rd. That these resolutions be placed upon the records of this Association, and a copy be sent to the family of the deceased, and published in two of the State medical journals.

R. R. Walker, M. D.,
Thos. Moody, M. D.,
Geo. S. Stell, M. D.,
Committee.

